# The Patient-Centered Approach in Rheumatologic Painful Diseases: A Narrative Review

**DOI:** 10.7759/cureus.22244

**Published:** 2022-02-15

**Authors:** Serge Perrot, Antonio Montero Matamala, Magdi Hanna, Giustino Varrassi

**Affiliations:** 1 Rheumatology, Cochin University Hospital, Paris, FRA; 2 Department of Surgery, University of Lleida, Lleida, ESP; 3 Pain Research, University of London, London, GBR; 4 Research, Paolo Procacci Foundation, Roma, ITA

**Keywords:** tramadol, dexketoprofen, drugs fixed dose combinations (fdc), multimodal analgesia, acute pain, patient-centered approach, rheumatologic painful diseases

## Abstract

A patient-centric approach to pain control represents a paradigm shift in analgesia and one that is both easy to endorse but challenging to execute. As pain mechanisms become increasingly elucidated, the understanding of pain has changed to encompass its complexities. Multiple types and mechanisms of pain have been described, and pain must be seen through the subjective experience of the patient. Earlier descriptions of pain based on intensity are one-dimensional and do not fully encompass the experience of pain. Thus, treating rheumatology patients or any patient in pain requires an understanding of the primary or secondary nature of the pain, underlying conditions, and patient factors such as anxiety, depression, fearfulness, and catastrophizing, all of which can shape and change the nature of the pain. Further, it is important to manage patient expectations concerning chronic pain as complete pain relief may not be possible, but a Patient Acceptable Symptomatic State (PASS) may serve. Functional goals are often more meaningful to patients than pain scores. Pharmacologic therapy for pain must consider side effects as well as analgesia. Patient-centered pain control requires a focus on wellness and disease prevention, personalized care plans, education, support for self-care, and may involve coordination across disciplines to help the patient meet personally meaningful objectives. While patient-centric care has become a buzzword in modern medicine, it is extremely relevant and may be very beneficial to pain patients.

## Introduction and background

While patient-centric healthcare has recently emerged as a desirable goal in modern medicine, patient-centered strategies to manage pain can be challenging and complicated to execute. The science and knowledge of pain are changing so rapidly that the definition of pain was revised in 2020 by the International Association for the Study of Pain (IASP). The new IASP definition states that pain is an unpleasant sensory and emotional experience associated with, or resembling that associated with, actual or potential tissue damage [1]. From the keynotes offered with this revised definition, it is important to view pain as inherently subjective, that is, a personal experience that is colored to varying degrees by biological, psychological, and social factors [1]. In this regard, pain must be made distinct from nociception, which is strictly a neurologic phenomenon. Moreover, pain cannot be inferred solely from sensory neuronal activity. The experience of pain is highly individual: people develop their ideas about pain over a lifetime within the contexts of their upbringing, familial situation, culture, and spiritual background [2]. What a patient reports as pain must be respected and not discounted or disbelieved, even when it seems to contradict clinical expectations. While certain forms of pain have an adaptive function, pain can become maladaptive and adversely affect function and well-being. Thus, treating pain in a patient-centered approach requires the clinician to appreciate the subjective experience as well as the physiologic aspects of pain.

## Review

Chronic primary musculoskeletal pain is defined as chronic pain localized in the muscles, bones, joints, and/or tendons that persists or recurs for >3 months and is associated with significant emotional distress or functional disability [3]. This recognizes chronic primary musculoskeletal pain as a disease in itself, classified as ICD-11. The IASP definition introduces several new and important concepts. First, it distinguishes between primary and secondary musculoskeletal pain. Chronic primary musculoskeletal pain must be viewed as a condition in its own right, while secondary musculoskeletal pain arises from some underlying disease or other condition, such as persistent inflammation, possibly caused by infection, structural damage, or neurological dysfunction. Second, by considering the subjective experience of musculoskeletal pain, it integrates the biomedical and psychosocial aspects of pain [4]. Thus, in patient-centered musculoskeletal pain care, the first step is to consider whether the pain is primary or secondary and, if the latter, what factors might be contributing to the pain. This is a major change from, for example, the World Health Organization’s “pain ladder” for treating cancer pain and common pain assessment tools such as the visual analog scale, which assess pain exclusively concerning its intensity [5]. Pain intensity remains an important consideration, but it does not entirely capture the pain experience. When clinically evaluating pain, it is first important to distinguish acute from chronic pain. In many ways, acute pain is simpler; it has a clear etiology and can be treated mainly by reducing its intensity. Chronic pain not only has different mechanisms but also incorporates a rich, multidimensional biopsychosocial experience. With chronic pain, etiology and intensity must likewise be identified, but the patient must also be assessed about how and how much pain interferes with function, the social impact of the pain, and how much psychological distress the pain causes. Particularly with rheumatologic diseases, dysfunction must be addressed and treated as much as a pain to restore the patient to greater well-being.

Pain mechanisms can be nociceptive, inflammatory, neuropathic, or nociplastic. In the case of nociceptive and inflammatory pain, such as the pain associated with rheumatoid arthritis, inflammatory processes are involved [6]. Neuropathic pain can be caused by neuronal damage; a good example is a post-surgical pain. Nociplastic pain recently identified and described in the literature, is due to abnormal signal processing in the central nervous system and may occur without neuronal lesions. Fibromyalgia is a type of nociplastic pain [7,8]. Pain mechanisms may occur singly or in combination (multimechanistic pain syndromes). In general, nociceptive and inflammatory pain are forms of acute pain, while neuropathic and neuroplastic pain are chronic forms; however, acute postsurgical pain may also have a neuropathic component [7].

The pharmacologic armamentarium for pain is large and diverse, to the point that it can be challenging to choose the appropriate pain regimen for individual patients. Pharmacological pain management may include opioids, steroid injections, antidepressants, anticonvulsants, nonsteroidal anti-inflammatory drugs (NSAIDs), anxiolytics, topical treatments, not to mention nonpharmacological means such as hot or cold therapy, occupational therapy, exercise, massage, and others. When making prescribing choices for pain patients, it is important to consider both pain control as well as side effect management. For example, opioids are effective pain relievers, but their adverse effects can be treatment-limiting [9], and there are concerns about potential misuse and abuse [10]. NSAIDs can be effective pain relievers but may not be suitable for patients at elevated risk for cardiovascular conditions [11]. Even paracetamol (acetaminophen) is associated with liver toxicity at supratherapeutic doses [12].

Low back pain (LBP) is a prevalent condition that is often described temporally as acute LBP (< 6 weeks), subacute LBP (6-12 weeks), and chronic LBP (> 12 weeks) [13]. While the temporal definitions are useful and easy to apply, the underlying pain mechanisms of these three types of back pain are fundamentally distinct. Acute LBP must be considered concerning etiology, as it may occur with trauma, malignancy, or other condition. Subacute LBP is particularly concerning as this indicates the patient is at risk for the development of chronic LBP, which can be much more challenging to treat.

While not all guidelines offer a consensus on the meaning of various “flags” [14], they can be important for effective treatment. Red flags are clinical features associated with a serious condition that necessitates urgent evaluation and treatment. Red flags may occur in acute LBP and be caused by tumors, infections, fractures, and neurological damage. If red flags are observed, the patient should be evaluated by the appropriate specialist(s) to get the necessary treatment as part of the overall treatment plan [15]. In the subacute phase, patients with yellow flags show evidence of certain prognostic factors that they may develop painful musculoskeletal conditions that could result in loss of function [16]. Among the yellow flags are unhelpful beliefs about pain or expectations of a poor outcome, negative emotional responses such as worry or fear but not rising to the level of a diagnosed mental health disorder, and potentially harmful pain coping strategies such as avoiding certain activities, not exercising, social isolation, and over-reliance on passive treatments such as analgesics [16]. When evaluating for yellow flags, it is important to assess the patient’s attitudes and beliefs about pain, behaviors, emotions, family situation, attitudes about work, and so on. For example, it may be helpful for the clinicians to ask patients about whether they think recovery is possible, whether they believe they can regain lost function, or if they are fearful that the injury might result in losing their job and descending into financial insecurity. There are questionnaires to help address yellow flags, but they can be time-consuming to administer. The Tampa Scale of Kinesiophobia evaluates the patient’s fear of movement in the context of musculoskeletal pain [17]. The Fear Avoidance Belief Questionnaire is related to kinesiophobia but involves the negative ramifications of certain maladaptive coping strategies that patients can develop [18,19].

Different specialists assess LBP in different ways, and these distinctions may vary even more by country. After checking for the presence of red flags (trauma, infection, tumor, and so on), rheumatologists and orthopedic specialists tend to look at the range of motion and reflexes, use multiple imaging technologies, and evaluate the patient using scales that assess pain and disability. On the other hand, pain specialists and neurologists may assess for neurological damage, employ neuropathic pain assessment tools, and make limited use of magnetic resonance imaging if imaging is used at all [20]. Rheumatologists and rehabilitation specialists focus more on functional restoration; neurologists look at neurological pain components; pain specialists treat the pain first and foremost. Local physical examinations are important for all types of musculoskeletal pain.

Catastrophizing, a stubborn and sustained expectation that the worst possible outcome will occur, is a cognitive behavior clinically associated with worse outcomes. Specifically, catastrophizing can lead patients to fear injury to the point of kinesiophobia, leading them to avoid movement, delaying their recovery, and increasing their risk of disability, possibly leading to depression. Catastrophizing can form a feedback loop where the expectation of disability provokes behaviors that support disability, rather than behaviors that confront the problem with positive expectations and activities aimed at recovery [21]. Functional goals, such as the ability to do chores at home, drive, go to work, garden, play sports, and so on, are often more meaningful to the patient than strictly clinical goals, such as achieving certain pain scores or metrics on exercise tests [22]. Clinicians may ask patients to name one or two domains in which their pain limits them: home, work, outdoor activities (including driving, shopping), sports, leisure, and social activities. Clinicians may then ask patients to identify which specific activities are of the highest importance to recover. Even a shortlist of one or two domains and a couple of activities can help set appropriate and meaningful functional goals. For instance, one patient may be more concerned about the ability to drive and do ordinary things around the home than participating in sports, although for another patient, the ability to return to playing sports may be the most motivating goal.

Patient-reported outcome measures are relevant patient-centered metrics that can help to measure progress for an individual patient. The Patient Acceptable Symptomatic State (PASS) is the level of pain intensity that a patient considers acceptable. Since complete pain relief is not a realistic goal for many chronic pain patients, the PASS metric allows the patient to set a personal standard. The PASS levels must be determined by the individual patient and will vary among patients [23]. Note that patient-reported outcomes in real-world clinical practice can differ markedly from patient-reported outcomes in clinical trials because real-world values are adapted to the patient’s lifestyle and activities [23]. The Patient’s Global Impression of Change (PGIC) defines for an individual patient what constitutes a clinically meaningful difference in pain intensity [24]. In clinical trials and practice, pain is often measured on a visual analog or numeric scale, which may show absolute or statistically significant changes that are not meaningful to the patient [24]. Unlike pain scales alone, the PGIC also incorporates how and to what extent adverse events might affect the patient, the overall tolerability of the drug regimen, and other aspects of care. For example, some pharmacological regimens may produce significantly lower pain intensity scores but with such disagreeable side effects that the patient does not find the treatment beneficial overall. Other aspects of care that patients may object to include pill burden, drug-drug interactions, expense, frequent clinic visits, and limitations of the drug regimen, such as avoiding sunlight or certain foods.

The Outcome Measures in Rheumatology meeting (OMERACT) found that the most meaningful patient-reported outcomes measured improvement, well-being, the onset of analgesic action, and durability of results rather than just pain intensity levels [25]. PASS scores and minimal clinically important improvement (MCII) have been established in a clinical trial but not among patients in their everyday lives. In a study of 2,414 hip or knee osteoarthritis patients (mean age 67.3 years) were evaluated for PASS and MCII over seven days. Scores in PASS and MCII were higher from the patients in everyday life than they were in the clinical trial. PASS for this cohort of four for pain at rest (79.4%) and five at movement (74.7%) was higher than PASS scores set forth by clinical trials in this population. Further, the MCII of -1 in real-world clinical practice was lower than found in clinical trials. Thus, values for acceptable pain and meaningful relief may differ markedly between clinical trials and clinical practice. These differences may be attributable to individual patient lifestyles, routines, and individual habits [23].

When treating pain, the first objective is to change the pain and reduce it. A clinically important improvement is generally considered to be a pain reduction of >50%. Although, improvement can be reported even when pain is reduced by >30%. Once that is achieved, a pain level is determined, pain treatment takes on a new objective: to maintain that state by reaching PASS, reducing disease activity, or other processes contributing to pain. As that objective is met, the next goal is to approach normal values as closely as possible.

Many guidelines and pain practice recommendations recommend and incorporate a patient-centric approach, allowing for treatment to be individualized for each patient [26]. The Osteoarthritis Research Society International (OARSI) for knee pain sets forth certain core treatments, such as weight management, land, and water exercise, strength training, and patient education for self-management. These core treatments can be adapted to the patient’s individual goals and objectives. Treatments can then vary depending on whether the osteoarthritis is limited to the knee only or extends to other joints and whether other comorbidities are present [26]. The European League Against Rheumatism (EULAR) has guidance for inflammatory arthritis and osteoarthritis based on a patient-centered approach and a biopsychosocial model, which involves assessments of the patient based on the patient’s needs, preferences, and priorities. This can evolve into a personalized pain management program using a shared decision-making model that may include: physical activity or exercise, orthotics, psychological or social interventions, sleep interventions, weight management, pharmacological treatment, and joint-specific management [27].

Technological advancements in the form of smart devices, interconnectivity, apps, biofeedback, telemedicine, and others, provide patients the opportunity to interact with a range of services from the privacy of their homes. Telemedicine allows patients to work remotely with physicians, paramedics, social workers, home health care, hospitals, those who practice traditional medicines, pharmacists, among many others. Technology can also help deliver education to patients, an important component of patient-centric pain care. In a study of 56 healthy adults who were divided into an interventional group receiving a 16-session education program and a control group who did not, the interventional group had significantly better performance in certain exercises (trunk muscle strength, spinal flexion range of motion, hamstring flexibility) and fewer visits to a physician for LBP in the next year [28].

Patient-centered care is an entirely new treatment paradigm both for clinicians as well as patients. It begins with a focus on wellness and disease prevention, personalized care plans, education and support for self-care, and coordination of care across disciplines. Patient-centered care must be convenient for the patient, personalized to meet individual and personally meaningful objectives, and measured based on improvements or setbacks rather than absolute scores.

## Conclusions

A patient-centered approach is particularly relevant in pain care. The first and most important foundation of pain care is assessment, which goes beyond the standard measurement of pain intensity to evaluate the pain in the context of the patient’s life. Patient assessment is the first step of pain treatment in this patient-centered model. Pain care demands that treatments offer clinically relevant benefits and personally meaningful goals to the patient. Patient-reported outcomes are crucial for patient-centric care. For optimal results, chronic pain patients must be actively involved in their treatment plans. This requires patient education, the commitment of both patient and clinical team, and a shared decision-making model.
